# Data-Driven Techniques for Detecting Dynamical State Changes in Noisily Measured 3D Single-Molecule Trajectories

**DOI:** 10.3390/molecules191118381

**Published:** 2014-11-12

**Authors:** Christopher P. Calderon

**Affiliations:** Ursa Analytics, Denver, CO 80212, USA; E-Mail: chris.calderon@ursaanalytics.com; Tel.: +1-720-663-9923

**Keywords:** single particle tracking, hierarchical Dirichlet processes, switching linear dynamical systems, measurement/localization noise effects, nonparametric Bayesian techniques, prior sensitivity

## Abstract

Optical microscopes and nanoscale probes (AFM, optical tweezers, *etc.*) afford researchers tools capable of quantitatively exploring how molecules interact with one another in live cells. The analysis of *in vivo* single-molecule experimental data faces numerous challenges due to the complex, crowded, and time changing environments associated with live cells. Fluctuations and spatially varying systematic forces experienced by molecules change over time; these changes are obscured by “measurement noise” introduced by the experimental probe monitoring the system. In this article, we demonstrate how the Hierarchical Dirichlet Process Switching Linear Dynamical System (HDP-SLDS) of Fox *et al.* [*IEEE Transactions on Signal Processing* **59**] can be used to detect both subtle and abrupt state changes in time series containing “thermal” and “measurement” noise. The approach accounts for temporal dependencies induced by random and “systematic overdamped” forces. The technique does not require one to subjectively select the number of “hidden states” underlying a trajectory in an *a priori* fashion. The number of hidden states is simultaneously inferred along with change points and parameters characterizing molecular motion in a data-driven fashion. We use large scale simulations to study and compare the new approach to state-of-the-art Hidden Markov Modeling techniques. Simulations mimicking single particle tracking (SPT) experiments are the focus of this study.

## 1. Introduction

Single-molecule experiments have a rich history in both life and physical science investigations [1,2,3,4,5,6,7,8,9]. Experiments capable of quantifying molecular motion with nanoscale resolution continue to be of interest to scientists and engineers (as partially evident by this Special Issue). The ability to experimentally quantify the motion of single-molecules without ensemble averaging has enabled researchers to gain various new insights about the kinetics of molecular interactions [10,11]. Single-molecule experiments typically produce a collection of “trajectories” (a time ordered sequence of force or position measurements) containing rich amount of temporal and spatial multiscale information; the desire to extract reliable quantitative information from these trajectories has inspired a variety of new computational algorithms, e.g., [12,13,14,15,16,17,18,19,20].

A surge of publications in optical microscopy techniques applied to monitor single-molecules in live cells [11,21,22,23,24,25,26,27,28] has generated much excitement because recent advances in optical imaging allow researchers to (relatively) noninvasively monitor biological molecules in their native environment. With both *in vitro* and *in vivo* single-molecule measurements, researchers must account for various complex features including inherent thermal fluctuations, inter- and intra-trajectory “heterogeneity” (induced by unresolved conformational degrees of freedom and/or a time changing micro-environment [29]), statistical artifacts introduced by the experimental apparatus, amongst other complications [10,11]. Quantifying the aforementioned “heterogeneity” to gain new insights on the system is often the motivation for carrying out a single-molecule study, but this feature of the data also severely complicates statistical analysis. For example, in current single-molecule studies, researchers typically only measure a point-like position of a fluorescently tagged molecule. Factors such as the molecule’s underlying conformation and/or if the tagged molecule is bound to another molecular complex in the cell tend to strongly influence the dynamics of position measurements, but these latent factors often cannot be directly observed in typical single-molecule experiments and need to be inferred from position versus time data (and the latent factors, or “kinetic states”, can vary substantially within and between trajectories).

In the earlier works, the spatial and temporal resolution afforded by the measurement device led researchers to focus mainly on Mean-Square-Displacement (MSD) type analyses to analyze single-molecule data [2,5,30,31]. MSD approaches have many undesirable features, namely they tend to introduce unnecessary temporal averaging (*i.e.*, they ignore the natural time ordering of the trajectory measurements) and they have a difficult time accounting for spatially varying forces (a common occurrence in live cells [29]). Advances in spatial and temporal resolution have inspired many researchers to develop new techniques for reliably extracting single-molecule level information out of measurements [12,13,14,15,16,17,18,19,20]. The previously cited works are most similar in spirit to the work presented, but all of the works encounter technical difficulties when there is an abrupt latent “state change” occurring in a molecule experiencing spatially dependent forces in a live cell environment (additional complications arise when position estimates are obscured by non-negligible “measurement noise”).

We demonstrate and discuss the utility of Hierarchical Dirichlet Process Switching Linear Dynamical System (HDP-SLDS) developed by Fox *et al.* [32] in identifying abrupt “state changes” where the number of states is unknown in advance, observations are corrupted by measurement noise, and the force or velocity field experienced by the molecule varies with position. An attractive novel feature of the HDP-SLDS approach is the joint estimation of the number of underlying latent states implied by the data along with kinetic parameter estimates. When estimating parameters, the likelihood function employed by the HDP-SLDS correctly accounts for the temporal and spatial statistical dependencies implied by a piecewise linear stochastic dynamical model. Other specific advantages over pre-existing approaches are discussed and illustrated through simulation examples motivated by Single Particle Tracking (SPT) experiments. Although we focus on simulations of SPT data, the basic idea behind the technique is anticipated to be applicable to a variety of single-molecule applications. In a companion paper, we illustrate how the technique can be applied to assist in the analysis of live yeast cells undergoing mitosis [33].

## 2. Methods

**Figure 1:**
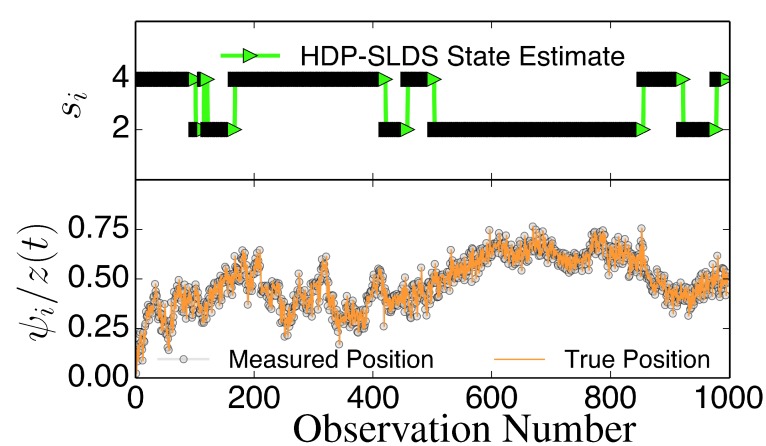
Illustration of abrupt changes in $\vec{µ}$. The bottom panel displays the *z* component of the unobservable state (orange solid line) and the noisily measured values *ψ_z_* (grey circles). The top panel illustrates the inferred states *s_i_*. In this trajectory, there are only two underlying states that differ in their $\vec{µ}$ value; an abrupt change in $\vec{µ}$ induced by a state change causes *z* to slowly drift towards a fixed point dictated by the (unobserved) $\vec{µ}$ associated with the state (the magnitude of the drift or “mean reversion rate” depends on the distance of *z* for the fixed point).

At a high level, the basic assumption underlying the HDP-SLDS method is that the dynamics of the particle measured can be approximated by linear stochastic differential equation (SDE) whose parameters are fixed for a contiguous block of time (or a “time window” [29]). When the HDP-SLDS algorithm declares that a “state change” has occurred, it implies that the algorithm has detected another contiguous block of observations exhibiting substantially different enough dynamics to declare that a new parameter vector is required to describe the dynamics (however, in the new block the dynamics are still assumed to be linear). The HDP-SLDS method of Fox *et al.* [32] is useful because it not only infers the number of unique parameters required to describe a single trajectory, but also temporally segments experimental trajectories into labeled states (each state has its own parameter vector characterizing the motion of the molecule). In other words, the HDP-SLDS algorithm not only identifies change points [15] but also labels the states (the algorithm permits for the possibility that the trajectory returns to a previously visited state). An illustration of the information provided by HDP-SLDS is shown in Figure 1; a discussion on the novelty of the HDP-SLDS over other methods using this example is provided after we present the details of the underlying discrete linear dynamical model associated with each state. In the text that follows the next subsection, we outline the main technical ideas underlying the HDP-SLDS introduced in [32].

### 2.1. Structure of the Fitted Linear Dynamical Model

For each unique state, it assumed that a discrete time series model of the form:

(1) $${\vec{r}}_{i+1}=\vec{\mu }+F{\vec{r}}_{i}+{\vec{\eta }}_{i};\;{\vec{\eta }}_{i}∼𝒩(0,\Sigma )$$

(2) $${\vec{\psi }}_{i+1}={\vec{r}}_{i+1}+{\vec{\epsilon }}_{i+1};\;{\vec{\epsilon }}_{i+1}∼𝒩(0,R)$$

can be used to describe the state dynamics. The position of the particle at time *ti* is denoted by the vector *r⃗*_*i*_ = (*x*_*i*_, *y*_*i*_, *z*_*i*_)^T^ and the measured value of the position at this same time is denoted by *ψ⃗*_*i*_ = (*ψ*_*x*_, *ψ*_*y*_, *ψ*_*z*_)^T^ (subscripts are used to index time); the position is not directly measurable due to “localization noise” and other artifacts induced by the experimental apparatus introducing “measurement noise” [13,29,34]. Measurement noise is modeled as a mean zero normal random variable with covariance R; the expression $\vec{\epsilon }∼𝒩(0,R)$ conveys that the random vector, *ϵ⃗*, is distributed according to the normal distribution 𝒩(0,R); the same notation is used for the discrete “process noise” vector $\vec{\eta }∼𝒩(0,\Sigma )$. The term $\vec{µ}$ represents the contribution of the time step multiplied by the average velocity vector of the particle. The matrix F accounts for changes in the velocity field as a function of the particle’s spatial position; random thermal fluctuations are modeled by *η⃗*. Note that each term in the equation above have units of length since discrete time series models have time integrated out. The unknown parameters characterizing discrete state-space representation are $\theta =(\vec{µ},F,R,\Sigma )$. Note that the HDP-SLDS presented in [32] assumed that all observations are uniformly spaced by ∆*t* time units; the approach also enforces a symmetric non-negative definite structure on Σ and R (allowing valid covariance matrices), and it allows F to be arbitrary (*i.e.*, no restrictions are made on the eigenvalues). However, in the data generating process discussed in Section 2.3, only F with “stable” eigenvalues are considered [35]; hence each state has a “fixed point” (*i.e.*, a well-defined average of value of a stationary distribution) associated with the state whose location is determined by $-F^{-1}\vec{µ}$. The magnitude of the eigenvalues of F determine the so-called “corral radius” or rate of “mean reversion” [19]. It should be noted that the parameters in the Appendix make the *z* component numerically close to a unit root [35] and can be considered a “pure diffusion” (*i.e.*, infinite corral radius) for the practical purposes of this work.

In Figure 1, we illustrate the subtle effects of $\vec{µ}$ switching randomly between two states (with all other SLDS parameters the same). Time correlation associated with relaxation to the new $\vec{µ}$ value is induced by the velocity field (spatial variation in the velocity field is modeled by the $F{\vec{r}}_{i}$ in Equation (1)). Explicitly accounting for the temporal statistical dependence induced by spatial variations in the velocity field as well as measurement noise are some features distinguishing the model above from previous SPT works attempting to automatically identify state changes [17,18]; these model features, in addition to the HDP-SLDS not requiring the user to select the number of state present in the trajectory (the number of states is inferred from the data), make the HDP-SLDS approach unique. Upon an abrupt switch in $\vec{µ}$, the fixed point associated with the linear dynamical system changes; in this example the fixed point is stable and it represents the mean of the stationary distribution associated with the SLDS. However, the change in mean level is not assumed to be instantaneous in contrast to other hidden Markov Modeling (HMM) models used in single-molecule analysis, e.g., [15]. The system is assumed to slowly evolve to the new fixed point (and molecular positions are substantially time correlated). When measurement noise is present (on top of thermal or “process” noise), identifying the precise time change of $\vec{µ}$ poses a difficult state estimation problem. The simulations studies presented demonstrate that the HDP-SLDS method can identify this type of subtle regime change amongst others that may be of interest to single-molecule studies.

### 2.2. Basic Assumptions of the Hierarchical Dirichlet Process-Switching Linear Dynamical System (HDP-SLDS)

In this section, we briefly review the basic idea and unique features of the HDP-SLDS at a high level; more technical comprehensive reviews of the HDP-SLDS technique are presented in [32,36]. The main advantage of the HDP-SLDS method is that the number of states does not need to be specified in advance by the user. The data determines the appropriate number of states (the method can account for an infinite number of latent states). Transition between the states are determined by the so-called “concentration parameters” used in the prior over the state transition matrices [32,36]. The technique also introduces a “sticky” parameter that encourages temporal state persistence in segmentation [32] (the “sticky” parameter is discussed further when we present the graphical model characterizing the HDP-SLDS). In addition, the technique exploits exact and computationally efficient closed-form likelihood expressions afforded by the SLDS models (this permits one to correctly account for things such as the temporal statistical dependence induced by spatially varying velocity fields and effects of measurement noise). Within the nonparametric Bayesian HDP-SLDS, the temporal dependence implied by the SLDS models “scores” trajectory segment’s dynamical similarity according to the so-called “base-measure” prior parameters [32,36].

**Figure 2:**
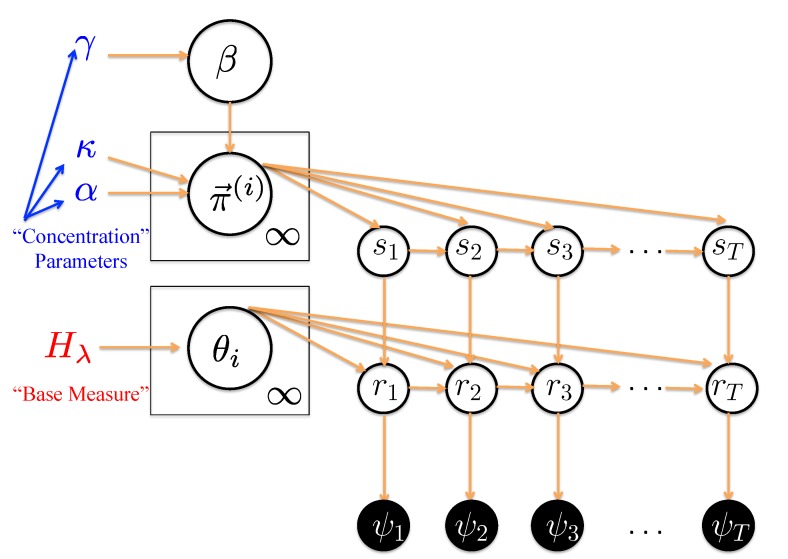
Graphical model [37] representation of the HDP-SLDS model using “plate notation” [38]. Unobservable (latent) random variables and system parameters denoted by open circles; observable quantities denoted by filled circles. The system state at observation taken at time *i* is denoted by *s_i_*, the position at this time is denoted by *r_i_*, and the measurement is denoted by *ψ_i_*. See text for description of HDP-SLDS model parameters. Arrows denote conditional dependencies in the graphical model.

Figure 2 displays the overall HDP-SLDS method in graphical model form [37] using “plate notation” [38] . Plate notation is the shorthand for a graphical model where rectangles or “plates” are used to group variables into a subgraph that repeat together; the number in the lower right-hand portion of the plate represents the number of repetitions of the subgraph in the plate. The observable random variables are denoted by filled circles and the unobservable (*i.e.*, latent) random variables and system parameters are contained in unfilled circles. The latent random variables are the states, position vectors, and measurement vectors at time *i*, denoted by *s_i_,* *r_i_,* *ψ_i_* (respectively). Hyperparameters [32], parameters assumed to govern the statistics of this hierarchical graphical model, are shown to the left of the plates. In the next paragraph we briefly describe the relevance of Dirichlet random variables, Dirichlet Processes (DP), Hierarchical Dirichlet Processes (HDP) and hidden Markov Modeling (HMM) used in the context of SPT [17]. This is followed by a brief discussion on the hyperparameters associated with the HDP-SLDS model.

In traditional HMM modeling, one assumes the number of states present before computing the likelihood of the HMM. More specifically, one makes an *a priori* assumption on *K* states being present and subsequently infers various probabilities associated with the *K* *×* *K* transition matrix along with the kinetic parameters characterizing each of the *K* states [17]. For each state *i*, the probability of transitioning is encoded in the transition vector, *π⃗*^(*i*)^, where *π⃗*^(*i*)^ ≡ (*π*_1_^(*i*)^, *π*_2_^(^*^i^*^)^, . . . , *π_j_*^(^*^i^*^)^, . . . , *π_K_*^(1)^); here *j* component *π_j_*^(^*^i^*^)^ represents the probability of transitioning from discrete state *i* to new state *j*. The finite collection of *K* transition vectors produces the transition matrix. Dirichlet random variables [17,39] are attractive to use as priors over the transition vectors since these random variables can be readily used to infer a vector whose finite set of discrete elements sum to one (a requirement for a valid transition vector) and Dirichlet random variables allow efficient Bayesian posterior computations due to the Dirichlet being a member of the exponential family [39].

In typical finite state HMM modeling, researchers must also prescribe a finite collection of *K* values to consider (this can be a difficult task in single-molecule experiments [29]) in addition to carrying out an *a posteriori* model selection criterion step to pick the “best” model amongst the collection of HMM models computed in a batch of separate HMM runs [17] (recall that each *K* value considered requires a new computation). However, it is possible to let the “data speak for itself” and infer the number of states along with the transition probabilities (as well as other parameters required to specify a dynamical model) in one computation by using a DP as a prior over the transition vectors (where an unbounded number of states are possible in the model). In the standard DP situation, where draws of the process are assumed to be generated using a continuous base measure (the situation encountered in SLDS), the probability of *π⃗*^(^*^i^*^)^ sharing states with *π⃗*^(^*^j^*^)^ is zero if *i* ≠ *j* [36,37]. The key to practical application of DP priors in SLDS modeling requires one to turn to Hierarchical Dirichlet Process (HDP) [37] since this hierarchical framework allows “state sharing” [36,37]. To construct an HDP, two DPs are drawn sequentially and the output of the first DP draw serves as the base measure to the second DP. Since a realization of a DP is discrete with probability one [39], this allows “state sharing” [37]. The “shared” transition vector is denoted by *β* in Figure 2; more specifically, this measure is modeled as being a draw from a Griffiths, Engen, and McCloskey (GEM) [36,37] process parameterized by *γ*. The number of active states (*i.e.*, the states with non-negligible probability) is determined by *γ*. “Weakly informative” hyperparameters are put over *γ* [32]. Subsequently, *β* is used as the probability measure governing the *π⃗*^(^*^i^*^)^’s, *i.e.*, *π⃗*^(^*^j^*^)^*∼* *DP*(*α*, *β*) where *DP*(*α, β*) denotes a Dirichlet Process characterized by probability measure *β* multiplied by the scalar scale parameter *α* (*α* plays the same role as *γ* in the GEM [37]).

Fox and co-workers expanded on the HDP by adding yet another feature important to SPT modeling. The HDP allows a “sharing of states”, however the DP prior over the transition vectors within the HDP framework does not have a mechanism for promoting state-persistence (*i.e.*, nothing stops self-transitions, quantified by *π_i_*(*i*), from being small in the standard HDP framework). State-persistence is usually believed to be common in many SPT applications. To encourage a model allowing state persistence, Fox *et al.* [32] introduced a “sticky parameter” *κ*. This parameter modifies the DP prior over the transition vect ${\vec{\pi }}^{\left(j\right)}∼DP(\alpha +\kappa ,\frac{\alpha \beta +\delta_{ij}}{\alpha +\kappa })$ where *δ*_*ij*_ is the Kronecker delta. The bias toward self-transitions is quantified by $\rho \equiv \frac{\kappa }{\kappa +\alpha }$. Within the original HDP-SLDS framework, a Γ(*a*, *b*) with hyperparameters *a* and *b* is placed over both *γ* and *α* + *κ* and a Beta(*c*, *d*) hyperprior with hyperparameters *c* and *d* is placed over *ρ*. The overall HDP-SLDS model is summarized in Figure 2. The number of hyperparameters that need selection may seem alarming in the HDP-SLDS, however as we will demonstrate later in the results section, the method is not overly sensitive to the concentration parameters; techniques for tuning the base measure parameters (represented by *λ*) are presented elsewhere [33].

### 2.3. Data Generating Process (DGP)

The original SLDs associated with the HDP-SLDS inference algorithm assumed a discrete stochastic model of the form shown in Equations (1) and (2). This facilitates plugging into the discrete Kalman filtering and smoothing equations [32,36], but it complicates physical interpretation of parameters (e.g., the analog of the “diffusion” matrix, Σ, depends on the observation frequency ∆). For the data generating process (DGP) used to simulate trajectories, we specify the parameters in a continuous stochastic differential equation (SDE) form [29]. The equations for mapping between the continuous and discrete time formulations is presented elsewhere [33].

The continuous time analog of Equation (1) is given by the following SDE:

(3) $$d{\vec{r}}_{t}=\Phi F({\vec{r}}_{t})dt+\sqrt{2}\sigma d{\vec{B}}_{t}\;$$

In the equation above, *F* ( *r⃗*) represents the effective force experienced by the particle located at position *r⃗*, Φ models the friction matrix, and *σ* is related to the diffusion coefficient, and *B⃗t* represents a standard multivariate Brownian motion (with the same dimension as *r⃗*) at time *t* [29]. This overdamped Langevin framework is fairly general, e.g., non-linear and/or time dependent forces can fit to data using this type of model [13,29,40,41]. Note that in the HDP-SLDS we use F to denote a fixed matrix, whereas in the overdamped Langevin equation above *F*(*r⃗*) is a vector depending on the current state.

In the specific linear parametric models considered in this article, each SDE contributing to an SLDS state (or “mode”) is parameterized by a finite dimensional vector denoted by θ. The parameters contained in θ and the remaining terms in Equation 3 are defined by the following equations:

(4) $$F(\vec{r})=B(r-\vec{r})$$

(5) σ=C

(6) Φ = *σσ*^T^*/k_B_T≡D/k_B_T*

In the expressions above, *k*_*B*_*T* represents Boltzmann’s constant multiplied by the system temperature. The collection of parameters to be estimated will be denoted by $\theta \equiv (\vec{r},B,C,R)$; a separate θ is estimated for each unique SLDS state. In the models considered throughout this article, $\vec{r}$ is a vector corresponding to the fixed point of the discrete model discussed earlier (*i.e.*, $\vec{r}=-F^{-1}\vec{µ}$). B, C, and R are real matrices. B determines the confinement or “corral radius” [19,31] and the “square” of C gives the diffusion matrix *D*, (*i.e.*, $D=CC^{\text{T}}$). The DGP identifying the states are expressed in terms of these physically interpretable parameters; an expanded discussion on the physical interpretation of the continuous time SDE parameters is presented in [29].

We allow any given trajectory to be a combination containing anywhere from two to four states. Measurements of each of the four states are corrupted by measurement noise characterized by a common R. We refer to “State 1” as the reference or base state and denote the parameters of this state using the subscript “*base*”; the dynamics of the first state are characterized by the following continuous time SDE parameters: *Dbase*, $B_{base}$, $r_{base}$. The specific values of the constants and parameters are reported in the Appendix. “State 2” occurs when *z < C*_1_ and *y* < *C*_2_ (in this state only the diffusion coefficient decreases from *D_base_* to *D_alt_* = *D_base_*/10; all other parameters are the same as “State 1”). “State 3” occurs when *z* > 0 and *y* > *C*_2_ (this is a “bound or confined” state where *D*_*base*_ changes to *D*_*alt*_ = *D*_*base*_*/*10 and $B_{base}$ to $B_{alt}$ and $r_{base}$ remains the same). Finally “State 4” occurs when *z* < 0 and *y_alt_* < *y* < *C*_2_; in this state only ${\vec{r}}_{base}$ changes to ${\vec{r}}_{alt}$ *≡* (*x*_*alt*_ , *y*_*alt*_, *z*_*alt*_)^T^ (all other parameters of “State 4” are identical to “State 1” so it is equivalent to a change in $\vec{µ}$ and the associated fixed point of the stationary process).

## 3. Results and Discussion

**Figure 3:**
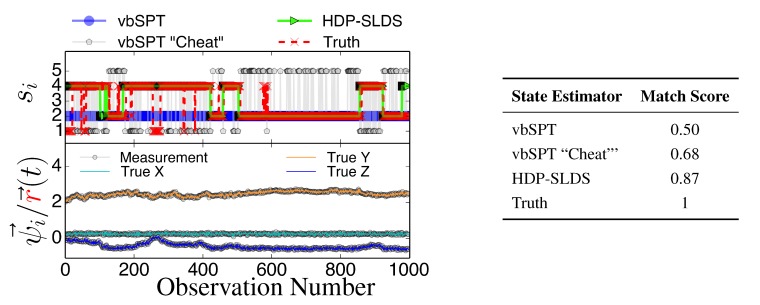
State estimates for three different state estimators (see text for description) along with the true state sequence (top panel), the 3D trajectory showing unobservable position and observable position (bottom panel), and the table quantifying the performance of the state estimators through the “Match Score”, which is defined as one minus the average Hamming distance for the trajectory.

**Figure 4:**
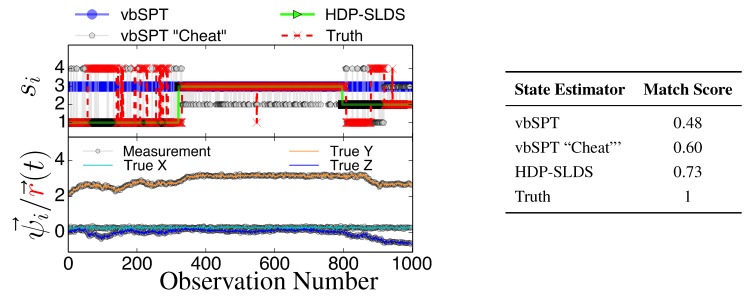
Same as Figure 3 except a new trajectory is analyzed where a different sequence of states is sampled.

Figure 3 and Figure 4 display two representative trajectories of $\vec{r}$, *ψ⃗* (bottom panel) along with the true/estimated state sequence (top panel). A table is provided to the right of the trajectory plots where the “Match Score” quantifying the quality of the HDP-SLDS [32] and vbSPT [17] state estimators applied to the displayed trajectory is reported. The “Match Score” is defined as equal to one minus the average Hamming distance and a “Match Score” of 1 denotes perfect performance. The Hamming distance indicates the sum of the number of correct state assignments; the average Hamming distance divides the sum by the length of the time series. Hence an average Hamming distance of 0 denotes a situation where the algorithm matched states precisely and 1 denotes a situation where not a single state was matched correctly. Recall that the vbSPT technique is a variational approximation to a classic HMM model; also note that the current publicly available software implementation of vbSPT does not account for all statistical effects induced by Gaussian measurement noise and vbSPT relies on post analysis model selection criteria to select the number of hidden states.

All estimators in Figure 3 and Figure 4 were provided priors having the mean diffusion coefficient and measurement noise parameters matching the DGP exactly. The technique labeled as “vbSPT” processed *ψ⃗* measurements directly (and used model selection criteria to find the best model containing 1–10 states) and that labeled “vbSPT Cheat” was carried out similarly, but the algorithm processed $\vec{r}$ directly (“Cheat” is used to label this estimator because in practice one cannot avoid measurement noise when analyzing laboratory data). The HDP-SLDS method estimated states by only analyzing a single long trajectory of *ψ⃗* containing 1000 observations. The vbSPT method was allowed to “pool” ten long trajectories (the collection provided an adequate representation of the four underlying states in the DGP) in an attempt to help this algorithm’s performance.

The HDP-SLDS method is able to quickly identify long lived state sequences, however it has the most difficultly in quickly identifying changes between State 1 and State 4 (where $\vec{µ}$ changes abruptly). Large scale simulations shown later quantify the transition between the various states more precisely. The “vbSPT” case consistently only estimates one state. However, it should be emphasized that the approach advocated in [17] was not designed to explicitly account for measurement noise, changing $\vec{µ}$ type parameters, or spatially varying forces. The vbSPT algorithm’s aim was to identify changes in diffusion coefficients in scenarios where measurement or localization effects are negligible in relation to the diffusion coefficient. The vbSPT technique was originally motivated to study a large collection of short SPT trajectories where it is not practical to estimate effective forces (in contrast to other SPT studies [29,33,42]).

Figure 3 and Figure 4 also illustrate how the approach labeled as “vbSPT Cheat” can identify the occurrence of state changes when measurement noise is removed in most situations, but in the situation studied, the “vbSPT Cheat” rapidly switches between two states for each single true state (rapid state switching is intentionally suppressed in the HDP-SLDS approach due to the use of “sticky” parameters [32]). We elected to compare the HDP-SLDS approach to vbSPT because this approach was most similar in spirit to the HDP-SLDS; the latter is better suited to long trajectories and the former is tailored to simultaneously analyzing a large collection of short trajectories (note: when measurement noise is not subtracted, the vbSPT method consistently estimated only one state in the scenarios studied despite 2–4 states being present in each trajectory).

For the remainder of this paper, we focus almost exclusively on the HDP-SLDS results since we aim to show its utility in extracting detailed information out of states representative of classic modes of motion [31,43] (*i.e.*, “directed diffusion”, “confined diffusion”, “pure diffusion”). Note that the “pure diffusion” case is technically a stationary process with very weak mean reversion. All results that follow analyze a fixed collection of 500 trajectories each containing 1000 uniformly spaced observations. The HDP-SLDS is applied to single trajectories (*i.e.*, trajectories are not pooled). In each run, prior parameters are altered, but the same set of 500 trajectories are analyzed/re-analyzed under different HDP-SLDS “tuning parameters”.

Table 1 displays the average Hamming distance (recall this number is between 0 and 1, with 0 denoting a perfect fit) observed in the population of 500 trajectories obtained after 10,000 Markov Chain Monte Carlo (MCMC) draws were generated to make state assignments. The runs labeled as “Baseline” use the known diffusion coefficient and measurement noise of the DGP as the mean of the inverse Wishart prior parameters used in the HDP-SLDS analysis; the case labeled *D/*4 divides the known average of the DGP and uses this as the average in the inverse Wishart prior over *D* (similarly for the measurement noise covariance, *R*). We also show the vbSPT results obtained when the exact DGP parameters are provided to the algorithm. (Recall that this algorithm was not tailored for this type of data and it consistently picks one state; however, the vbSPT technique is the most similar approach to the HDP-SLDS commonly currently used by the SPT community in the author’s opinion.) As can be readily observed (and as stated in [36]), the base measure parameters can strongly influence the state segmentation inference and a “properly tuned” HDP-SLDS state estimator can have impressive performance in detecting subtle changes in trajectories containing spatially dependent forces, thermal noise, and measurement noise. Fortunately, tools exist for approximating trajectory-wise statistics on 2D and 3D trajectories [29] (such tools can be used to construct data-driven priors and base measure parameters; however this topic is covered elsewhere [33]). Table 2 confirms that varying the primary “concentration parameters” associated with the HDP-SLDS [32] has little effect on the state segmentation results.

**Table 1:** Effects of misspecifying “Base Measure” parameters. The average Hamming distance (a number between 0 and 1, with 0 indicating a perfect match) measured over 500 trajectories each of length 1000 (empirical standard errors indicated in parenthesis). The cases in the leftmost column are described in the text.

Case	Hamming Dist.
Baseline	0.16 (0.03)
*D*/4	0.31 (0.04)
*R*/4	0.39(0.04)
vbSPT	0.28 (0.04)

**Table 2:** Effects of misspecifying “Concentration Measure” parameters containing same information as in the previous table.

Case	Hamming Dist.
Baseline ( *γ_b_* = 0.01; *ρ_c_*= 25)	0.16 (0.03)
*γ_b_* = 0.001	0.17 (0.03)
*γ_b_* = 0.1	0.15 (0.03)
*ρ_c_* = 100	0.16 (0.03)
*ρ_c_* = = 5	0.18 (0.03)

**Figure 5:**
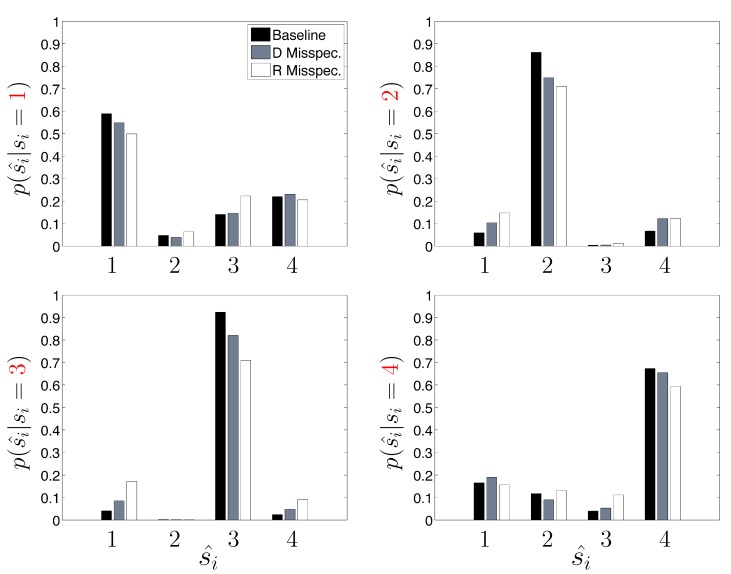
A finer breakdown of the HDP-SLDS performance as a function of the known underlying state for three different conditions studied in Table 1. The y-axis shows the empirical conditional probability of the state estimate *ŝ_i_* (x-axis) conditioned on the true (known) underlying state *s_i_* (the panels vary over the four truth states used by the simulated data generating process).

Next, we take a closer look at the error committed by the three HDP-SLDS analyses shown previously when trying to identify the four latent states used by the DGP (Table 1 reported only the overall average Hamming distance). In Figure 5, the empirical probability of state assignment (using the three HDP-SLDS methods used in Table 1) is computed using the known underlying state of the DGP. Previously, in Figure 4, we qualitatively demonstrated that abrupt and transient changes in $\vec{µ}$ were difficult to identify (*i.e.*, see transitions from State 1 to State 4 and back occurring near observations 1–250). This is because the process mean changes quickly, but the position (and hence measurement) takes time to adjust to the new mean location (or the new “energy well minimum” if one wants to use the harmonic spring analogy) and the inference algorithm needs to accumulate sufficient evidence before it declares the existence of a new state. Figure 5 quantifies this phenomenon more accurately using a large population of trajectories. Abrupt changes in the diffusion coefficient (State 3) and confinement parameters (State 4) are more readily correctly identified by the HDP-SLDS algorithm. This plot also gives a finer grained picture of how an “improperly tuned” prior quantitatively affects state estimation.

**Figure 6:**
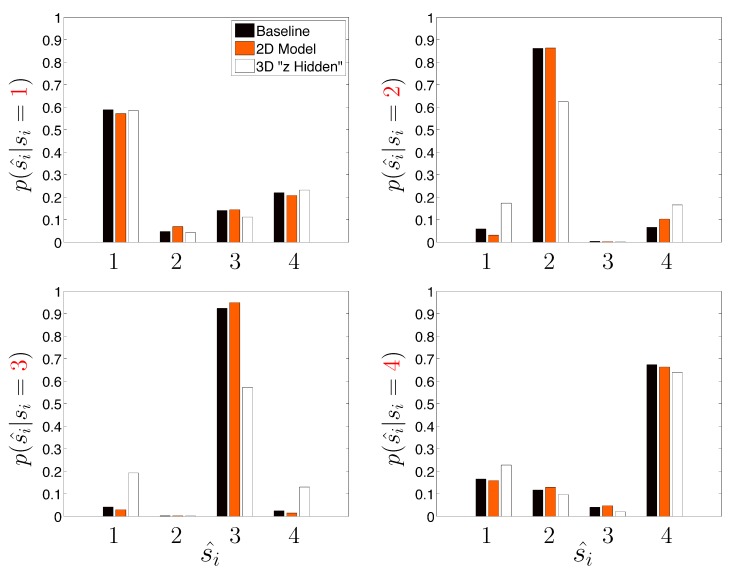
Same as Figure 5, except that effects of dimensionality of the underlying state model are investigated (see description in text).

Table 3 assumes that the DGP used for the priors are known precisely (an admittedly unrealistic situation) and re-analyzes the same set of 500 trajectories, except this time the algorithm is only presented in *x* and *y* measurements. The axial *z* dimension is considered unobserved (*i.e.*, the only available data is a time ordered sequence of paired *ψ*_*x*_ and *ψ*_*y*_ measurements); this situation is commonly encountered in SPT. However, recent advances in optical microscopy show promise in more accurately measuring long 3D trajectories [21,25,27,43]. The approach labeled “Naive 2D Model” considers the state to be a two-dimensional vector (*i.e.*, effects of *z* are not explicitly computed in the likelihood function of the HDP-SLDS) and the approach labeled “3D Model (Hidden *z*)” considers a Kalman filter where there is a three-dimensional state vector but the observation process is two-dimensional. Note how the “Naive 2D Model” slightly improves on the “Baseline” case in terms of the average Hamming distance. The reduction in dimension of the parameter vector characterizing the base measure governing the stochastic model improves the joint state and kinetic parameter inference in the scenario studied. A somewhat surprising result is the unambiguous statistically significant degradation in state segmentation obtained when the effects of *z* were attempted to be accounted for in by the state space model. The fact that there were no off-diagonal terms in F and Σ account partially for the strength of the degradation, but we include this example to show that “more is not always better” (*i.e.*, attempting to explicitly model known, but unobservable, coordinates can be potentially detrimental to state segmentation results). Figure 6 shows results analogous to Figure 5 for the “Naive 2D Model” and “3D Model (Hidden *z*)” model cases studied.

**Table 3:** Effects of model’s state dimensionality. The average Hamming distance (a number between 0 and 1, with 0 indicating a perfect match) measured over 500 trajectories each of length 1000 (empirical standard errors indicated in parenthesis).

**** **Baseline**	0.16 (0.03)
**“Naive” 2D Model**	0.12 (0.03)
**3D Model (Hidden ** ***z*)**	0.46 (0.04)

## 4. Conclusions

We demonstrated how the HDP-SLDS method can model simulations mimicking 3D single-molecule data. The technique was demonstrated by analyzing a large collection of long control simulation trajectories containing a varying mix of classical SPT “modes of motion” [2,5,44] as well as more difficult to detect changes (e.g., abrupt change in the spatial location of a “harmonic well-minimum” where statistical time correlation in the relaxation to the new harmonic well-minimum is non-negligible). Parameters selected were motivated by studies of transmembrane protein kinetics in the primary cilium [42]. It was shown that the HDP-SLDS framework can systematically account for spatially varying forces, the statistical effects of measurement noise, and an *a priori* unknown number of underlying latent states where other methods encountered problems due to neglecting key statistical features or making unnecessary approximations. The HDP-SLDS can obtain state-of-the-art segmentation results using only a single “long” trajectory (*i.e.*, one containing many time samples). For situations where there is benefit to pooling information from multiple long trajectories, alternative approaches similar in spirit to the HDP-SLDS show promise in single-molecule analysis [45]. The HDP-SLDS and other nonparametric Bayesian approaches extracting information from long time ordered sets of measurements [45] are nice complements to the technique of Persson *et al.* [17], which aims at pooling kinetic information from multiple short trajectories to identify the number of states. However, it should be noted that in the analysis of single-molecule data, a small finite set of discrete states describing a trajectory (or groups of trajectories) may not always be an appropriate representation of data measured in complex heterogeneous environments [29]. In cases where a small set of discrete SLDS states (driven by standard diffusive noise) can be informative about the underlying single-molecule system and one has “long” trajectories, the HDP-SLDS approach is useful because it is capable of producing accurate state estimation and temporal segmentation when compared with other state segmentation routines used in SPT data analysis. The HDP-SLDS approach also provides a systematic framework for the “time window” selection problem mentioned in [29]. Note that the HDP-SLDS method has been successfully applied to experimental SPT trajectories containing as few as 150 observations uniformly sampled at 22 frames per second [33].

Despite the fact that the HDP-SLDS technique is labeled as a nonparametric Bayesian method, we demonstrated that the parameters characterizing the base measure can still heavily influence state estimation and segmentation results (we also presented results confirming that sensitivity to the concentration parameters and hyperparameters is minimal [36] in the situations studied). The “nonparametric Bayesian” monicker attached to the HDP-SLDS is slightly misleading since the base measure depends heavily on an SDE model with an SLDS parametric structure; the model also has priors depending on a parametric structure. Prior parameter sensitivity is not unique to the HDP-SLDS approach; priors and hyperparameters affecting algorithm performance is typically common amongst Bayesian approaches [15,17]. Other approaches that are closer to a “nonparametric” spirit are potential alternatives (e.g., anomalous and standard diffusion driven models can be considered as in [18]), but such methods can encounter technical difficulties when faced with trajectories where velocity or forces are spatially dependent and the measured signal contains inherent “thermal noise” as well as measurement noise.

If accurate quantitative information about single-molecule trajectories are not available *a priori* (a common situation in single-molecule analysis), techniques for extracting data-driven base measure and priors parameters in a “single-molecule fashion” can be considered (see a companion manuscript [33]). Note also that goodness-of-fit testing can be leveraged to assess the fundamental HDP-SLDS assumptions against data without “ground truth ” available [29,33]; this feature is useful since in the analysis of live cell experimental data, one does not typically have the luxury of “ground truth”. In such situations, it becomes important to determine if there is adequate statistical evidence in the data to justify one segmentation over another. After a good segmentation is believed to be in hand, one can then attempt to refine parameters estimates characterizing the motion of the single-molecule trajectory [33]. Hence using nonparametric Bayesian ideas (such as the HDP-SLDS) along with frequentist ideas (such as those in [29]) shows great promise in reliably extracting new quantitative information from single-molecule data [33].

## Appendix (Simulation Parameters)

The basic DGP parameters used for the simulation defining State 1 referenced in Section 2.3 are as follows (implicit units: length [*µm*], time [*s*], force [*pN*]):

$$R_{base}=\left(\begin{array}{ccc} 0.04^{2} & 0 & 0\\ 0 & 0.04^{2} & 0\\ 0 & 0 & 0.04^{2} \end{array}\right),\;D_{base}=\left(\begin{array}{ccc} 0.01^{2} & 0 & 0\\ 0 & {\frac{10\pi }{180}}^{2} & 0\\ 0 & 0 & {\frac{10\pi }{180}}^{2} \end{array}\right),\;B_{base}=\left(\begin{array}{ccc} -40 & 0 & 0\\ 0 & -0.2 & 0\\ 0 & 0 & -1\times 10^{-9} \end{array}\right)$$

For the other states, the above are used along with $D_{alt}=\frac{D_{base}}{10}$, $B_{alt}=B_{base}$ except $B_{base}(2,2)=-10$ (allowing binding in *y*) and ${\vec{r}}_{base}=\left(\begin{array}{c} 0.25\\ \pi \\ 0 \end{array}\right)\;{\vec{r}}_{alt}=\left(\begin{array}{c} 0.25\\ \frac{13\pi }{18}\\ 0 \end{array}\right)$. For the state switching constants we use $C_{1}=\frac{30\pi }{180}\text{ and }C_{2}=\frac{165\pi }{180}$.

The simulation parameters above were motivated by studies of membrane diffusion in the primary cilium taken at physiological conditions (*T* = 310*K*) [42]. Note: the plots of $\vec{r}$ and *ψ⃗* presented in the main text were converted from “angle-like” coordinates to Cartesian *xy**z* by multiplying $\vec{r}$ and *ψ⃗* by $\frac{180}{\pi }$.
