# Correction: Calderon, C.P. Data-Driven Techniques for Detecting Dynamical State Changes in Noisily Measured 3D Single-Molecule Trajectories. *Molecules 19*, 18381-18398

**DOI:** 10.3390/molecules20022828

**Published:** 2015-02-09

**Authors:** Christopher P. Calderon

**Affiliations:** Ursa Analytics, Denver, CO 80212, USA; E-Mail: chris.calderon@UrsaAnalytics.com; Tel.: +1-720-663-9923

The author wishes to make the following corrections to paper [1] (doi:10.3390/molecules191118381, website: http://www.mdpi.com/1420-3049/19/11/18381):

(1) The fixed point reported on pgs. 18384 and 18388 corresponds to a different parameterization of the model reported Equation (1). That is, −*F*^−1^$\vec{\mu }$ corresponds to the fixed point of ${\vec{r}}_{i+1}$ = ${\vec{r}}_{i}$ + $\vec{\mu }$ + *F*${\vec{r}}_{i}$ (random noise components omitted). For the model in Equation (1), the correct fixed point expression is (*Id* − *F*)^−1^$\vec{\mu }$ where *Id* denotes the identity matrix. Accordingly, to be consistent with the discrete model shown in the published manuscript, the expression −*F*^−1^$\vec{\mu }$ on pgs. 18384 and 18388 should be replaced by (*Id* − *F*)^−1^$\vec{\mu }$. This was a typographical error and did not affect the results presented.
(2) The following information should have appeared in the Appendix, but was unintentionally omitted in the published version: *“The simulations were generated by using the Euler-Maruyama integration scheme using a time step size of* 2 × 10^−4^[*s*]. *The time series data was recorded uniformly in time with* ∆*t* = 1 × 10^−2^ *time units separating observations. Simulated measurement noise was then added to the time series to produce measurements. The HDP-SLDS toolbox currently available on Emily Fox’s webpage (http:// www.stat.washington.edu/ ~ebfox/ software/ HDPHMM_HDPSLDS_toolbox.zip) was used to implement the HDP inference.”*
(3) The last sentence in the published Appendix, *“Note: the plots ... by* $\frac{180}{\pi }$*.”* (pg. 18395), should not have appeared in the published work. The comment is not applicable to the final published version of the paper and adds confusion. The plots presented in the published manuscript did not use the ad hoc linear scaling discussed.

The following minor typographical errors were also identified post publication by the author:

On pg. 18384 *“... well-defined average of value of a stationary ...”* should be replaced by: *“... well-defined vector defining the mean value of a stationary multivariate ...”*

On pg. 18387 *“observation frequency* ∆*”* should be replaced by: *“time between observations, i.e.,* ∆*t”*

On pg. 18395 *B*_*base*_(2, 2) = −10 should be replaced by: *B*_*alt*_(2, 2) = −10

Due to axis mislabeling, replace:

**Figure 1:**
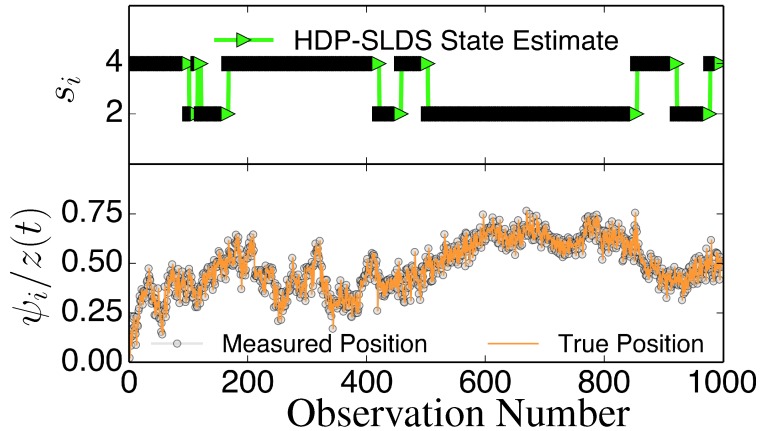
Illustration of abrupt changes in $\vec{\mu }$. The bottom panel displays the *z* component of the unobservable state (orange solid line) and the noisily measured values *ψ*_*z*_ (grey circles). The top panel illustrates the inferred states *s*_*i*_. In this trajectory, there are only two underlying states that differ in their $\vec{\mu }$ value; an abrupt change in $\vec{\mu }$ induced by a state change causes *z* to slowly drift towards a fixed point dictated by the (unobserved) $\vec{\mu }$ associated with the state (the magnitude of the drift or “mean reversion rate” depends on the distance of *z* for the fixed point).

with

**Figure 2:**
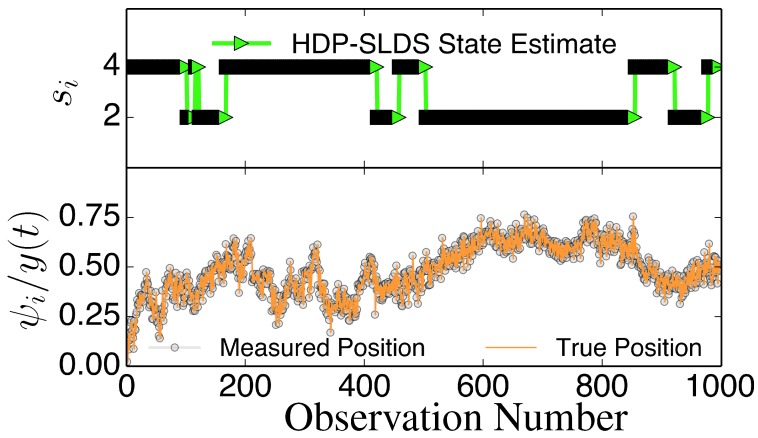
Illustration of abrupt changes in $\vec{\mu }$. The bottom panel displays the *y* component of the unobservable state (orange solid line) and the noisily measured values *ψ*_*y*_ (grey circles). The top panel illustrates the inferred states *s*_*i*_. In this trajectory, there are only two underlying states that differ in their $\vec{\mu }$ value; an abrupt change in $\vec{\mu }$ induced by a state change causes *y* to slowly drift towards a fixed point dictated by the (unobserved) $\vec{\mu }$ associated with the state (the magnitude of the drift or “mean reversion rate” depends on the distance of *y* from the fixed point).

These changes were only typographical corrections identified post publication by the author (the correct expressions were used in computations) and have no impact on the conclusions of the paper. The author would like to apologize for any inconvenience caused to the readers by these changes.

## References

[1] Calderon C.P. (2014). Data-Driven Techniques for Detecting Dynamical State Changes in Noisily Measured 3D Single-Molecule Trajectories. Molecules.

